# Cataract Surgery in Congenital Colobomatous Microphthalmia Associated With Intraorbital Cyst in an Adult

**DOI:** 10.1155/crop/6625168

**Published:** 2025-11-10

**Authors:** Yun-e Zhao, Fan Zhang, Dandan Wang

**Affiliations:** ^1^National Clinical Research Center for Ocular Diseases, Eye Hospital, Wenzhou Medical University, Wenzhou, China; ^2^Eye Hospital of Wenzhou Medical University Hangzhou Branch, Hangzhou, Zhejiang, China

**Keywords:** congenital catacract, intraorbital cyst, magnetic resonance imaging, microphthalmia, scleral defect, small corneal

## Abstract

We report a challenging congenital cataract surgery in an adult case of colobomatous microphthalmia associated with intraorbital cyst.

## 1. Introduction

Microphthalmia, characterized by a small eye in the orbit, is estimated to have a prevalence of 3–14 per 100,000 individuals [1–3]. Complex microphthalmia is associated with ocular disorders. Furthermore, colobomatous microphthalmia is a developmental anomaly that results from defective closure of the embryonic fissure at an early stage [4]. Therefore, it is often accompanied by other congenital eye malformations. Herein, we report a challenging congenital cataract surgery in an adult case of colobomatous microphthalmia concomitant with intraorbital cyst.

## 2. Case Presentation

A 30-year-old man was diagnosed with microphthalmia and congenital cataracts since childhood. As long as the patient could remember, he could not see with his left eye and could only see the silhouette of the person in front of him with his right eye; however, he was capable of taking care of himself. However, over the past 5 years, he complained of gradual deterioration of visual acuity in the right eye. In recent years, he was barely able to distinguish light from dark. He had previously sought medical attention elsewhere to no avail, and finally visited our institution. He allowed personal data processing and informed consent was obtained from all individual participants included in the study.

He had no history of systemic illnesses. He was delivered via normal delivery at term with an uneventful antenatal and perinatal period; developmental milestones for the patient's age were achieved. His mother had a similar condition with poor vision in both eyes since childhood but had not undergone medical examination. Except for his mother, the patient did not have any significant genetic or psychosocial family history.

Ocular examination revealed the best-corrected visual acuity of finger count before the eye (FC/BE) in the right eye and no light perception in the left eye. The patient had right-sided esotropia and horizontal end-gaze nystagmus. An external examination revealed microphthalmia, which was more severe in the left eye than in the right, with corneal diameters of approximately 3 and 6 mm in the left and right eyes, respectively. Slit-lamp examination revealed a flat corneal, a shallow anterior chamber, iris posterior synechia, and a cloudy lens (C3N3, LOCS II classification) in the right eye. A direct reflection to light was present in the right eye. Additionally, corneal edema with a flap anterior chamber and advanced cataract (C3N4) was observed in the left eye. Intraocular pressure (IOP) was 29.5 mmHg in right eye and undetectable in left eye (iCare-pro, ICARE, Finland). A detailed fundus examination could not be performed because of nystagmus and cataract.

## 3. Examinations

**Figure 1:**
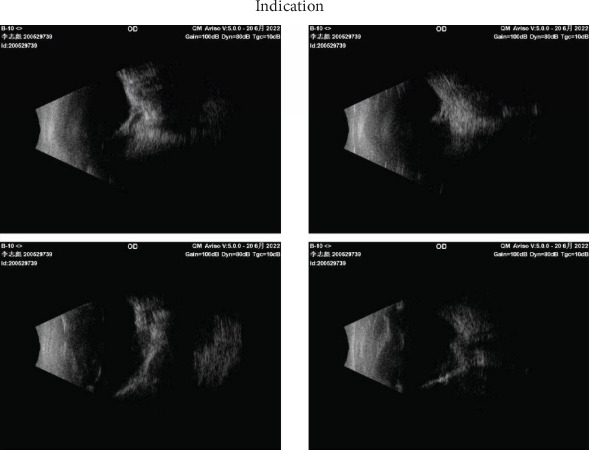
B-scan ultrasound showing a wide range of non-echoic areas behind the eyeball in right eye.

**Figure 2:**
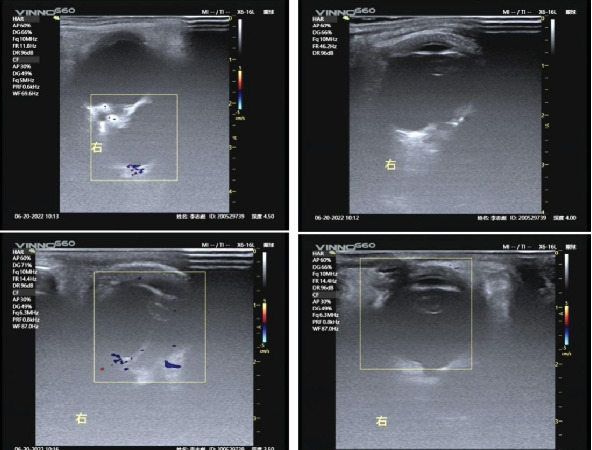
Doppler ultrasonography findings of the right eye. A large anechoic area is detected behind the wall of the eyeball, around which blood flow signals are detected.

A B-scan (AVIOSO, Quantel Medical, France) demonstrated that the structures of both eyes were disordered (Figure 1). In the right eye, the vitreous cavity was detected as a band of stiff echoes locally connected to the irregular wall of the eyeball; the location of the optic nerve could not be determined. A wide range of anechoic areas were detected behind the eyeball. Doppler ultrasonography of the right eye revealed a large anechoic area behind the wall of the eyeball, around which blood flow signals could be detected (Figure 2). Additionally, it revealed possible scleral and choroidal defects in the posterior wall of the eyeball, which appeared to communicate with a retrobulbar cyst. The axial length of the right eye was approximately 17.84 mm, the anterior chamber depth was 2.33 mm, the lens thickness was 3.34 mm, as measured using an optical biometer (IOLMaster 700, ZEISS, Germany). In the left eye, the vitreous cavity was detected as a mass of echoes with an irregular wall morphology, and posterior echogenicity was locally irregular. The axial length of the left eye was 11.8 mm, as estimated using B-scan ultrasonography. Due to the opacity of the visual axis area, the difficulty of fixation, the above results are reported to have a large standard deviation, and the corneal curvature could not be measured.

Visually evoked potential (VEP) was performed to assess visual function. It revealed the amplitude of P2 wave decreased in both eyes, and the waveform was not obvious.

**Figure 3:**
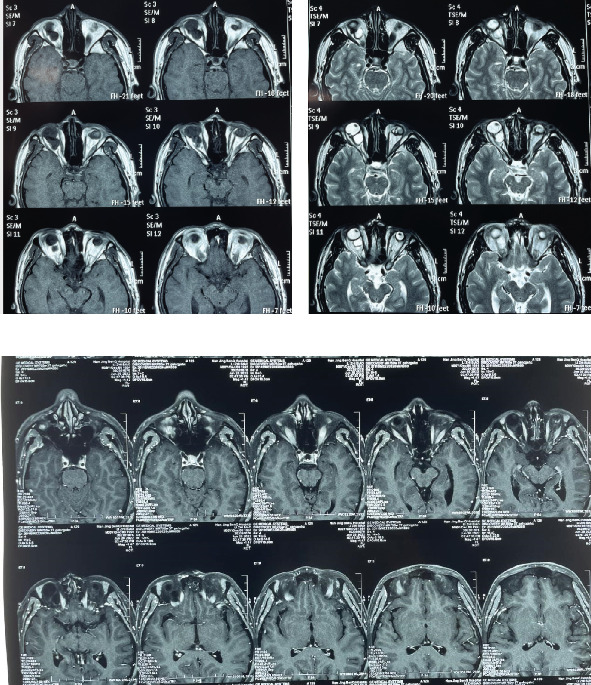
(a) Ordinary magnetic resonance imaging (MRI) of the orbit showing an irregular, well-defined mass measuring 13 × 9 mm, between the posterior inferior rectus muscle and the optic nerve in right eye. The mass compressed the optic nerve and displaced it to the nasal superior. The mass was isointense on T1-weighted images and hyperintense on T2 images. No communication between the bulbus oculi and the mass can be seen in the MRI findings. (b) Contrast-enhanced magnetic resonance imaging findings showing a fluid structure surrounded by a thin-walled cystic cavity.

Magnetic resonance imaging (MRI) revealed an irregular, well-defined mass measuring 13 mm × 9 mm between the posterior inferior rectus muscle and the optic nerve in the right eye. The orbital cyst exerted a mass effect on the optic nerve, displacing it into the superior nasal region. The mass was isointense on T1-weighted images and hyperintense on T2-weighted images (Figure 3).

Based on these findings, the following diagnoses were made: congenital cataract, microcornea, microphthalmia in both eyes, intraorbital cyst in the right eye, scleral defect in the right eye (suspected), esotropia, and nystagmus.

The patient's mother also underwent eye examination. She was diagnosed with congenital choroidal defects, congenital cataracts, iris defects, microcorneas, bilateral nystagmus, and posterior lens dislocation in the left eye. There was a similarity in the appearance of both eyes. Peripheral blood samples were obtained with the patient's consent for whole-exome high-throughput sequencing. One variant of RARB gene (NM_000965.5:exon2:c.241T>G:p.C81G) was detected, which may be related to the clinical phenotype. The above variants may cause microphthalmia syndrome type 12 (MIM: 615524).

## 4. Management

Considering that blurred vision might have been closely related to progressive cataracts and partially due to optic nerve compression, cataract surgery of right eye could potentially restore vision. Owing to the small diameter of the cornea, the lens diameter was speculated to be relatively small, conventionally used intraocular lens (IOL) may not be appropriate in power and size. Given the lack of ocular pain and proptosis, cyst excision was not recommended. After a comprehensive evaluation of the risks and benefits of surgery and detailed communication with the patient, a decision was made to perform cataract surgery in the right eye.

The corneal K value was set at 43.5 D, and IOL power was calculated according to the rough AL and the set K value; 50 D was required to achieve emmetropia According to Barrett Universal II formula, we prepared a highest power IOL(36D) available. Moreover, both the patient and us have reached a consensus that IOL implantation might not be possible because of small capsular bag.

## 5. Surgical Procedure

**Figure 4:**
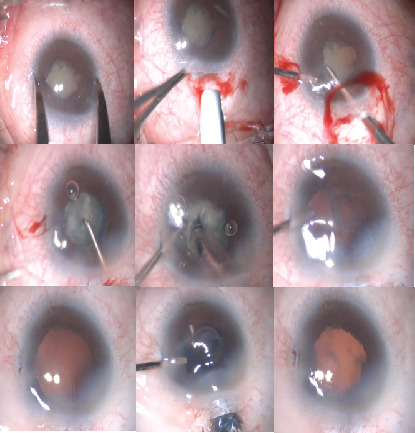
Cataract extraction with implantation of an intraocular lens in the right eye.

Surgery was performed by Dr. Zhao under general anesthesia, using the CENTURION system (Alcon Laboratories, Inc., United States). A 2-mm superior scleral tunnel incision was made due to the small cornea. After isolation of the iris posterior synechiae with an ophthalmic viscoat device, a 4.5-mm continuous curvilinear capsulorhexis was performed. The bottle height was first set at 70–80 cm, with the vacuum 300 mmHg and the flow rate 30 mL/min, to avoid posterior choroid herniation due to a possible scleral defect. However, bulky and dense nucleus was observed, and the efficiency was too low with the preset parameters. Therefore, the bottle height was appropriately increased to 85 cm, with the vacuum of 450 mmHg, and the aspiration rate 30 mL/min. The nucleus was successfully removed using phaco-chop technique. After aspiration of the cortex, a 13-mm-diameter one-piece foldable IOL (Proming A1-UV, Eyebright Medical Technology, China) was smoothly implanted (Figure 4, Supplementary video (available here)).

## 6. Outcome and Follow-up

**Figure 5:**
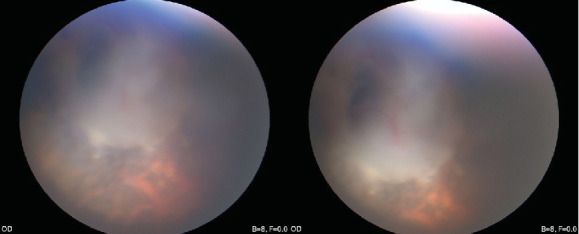
Fundus exploration during the operation.

The next day and 1 week postoperatively, the visual acuity of the right eye was FC/10 cm, FC/30 cm; however, recognition was slow. Although the patient's postoperative improvement was limited in terms of objective visual acuity, his quality of life improved significantly. One year after surgery, the visual acuity was FC/40 cm. From the two videos of vision examinations, he was observed to respond much quicker 1 year later. The patient no longer required a cane to walk and could distinguish between traffic signals, walk alone, and jog in a relatively empty park. The patient experienced true vision improvement in his daily life (Supplemental Figure 1). Additionally, a relatively clear fundus image was obtained, which indicated scleral defects in the optic disc (Figure 5, Supplemental Figure 2).

## 7. Clinical Discussion

Herein, we report a rare case of colobomatous microphthalmia with intraorbital cyst. The term microphthalmia is used to describe an eye with an axial length shorter than 20.5 mm or 18.5 mm in adults [5, 6]. Microphthalmia may also be associated with other ocular disorders, in which case it is termed complex microphthalmia. These ocular disorders may affect the anterior (e.g., Peters' anomaly) and/or posterior segments (e.g., persistent hyperplastic primary vitreous and retinal dysplasia). However, there were few reports regarding orbital cysts [4, 7–9].

Our patient's axial length of the right eye was about 17.84 mm. Cataract surgery in microphthalmos is associated with higher rates of complications and poorer visual outcomes. The most concerning intraoperative risk was the potential for uveal effusion or choroidal hemorrhage, most commonly precipitated by acute intraocular pressure decompression and subsequent malignant glaucoma [6, 10]. However, we considered that the presence of possible posterior scleral or choroidal defects may decrease the risks of uveal effusion or choroidal hemorrhage. Although VEP does not have a decent a- and b-wave, he was capable of taking care of himself 5 years ago. The patient's strong desire for surgery aided the decision. It turned out that although this surgery was full of challenges, the outcome was good.

The patient had a flat corneal with a diameter only 6 mm. This abnormal anatomical structure resulted in a shallow anterior chamber and limited surgical space. Intraoperative water leakage from the incisions could result in a poor surgical field due to the small and flat cornea, making the surgery a great challenge. Furthermore, there may be an extremely small lens size. Fortunately, the patient has a large lens size with a bulky and dense nucleus. An IOL of normal size was smoothly implanted into the capsular bag. It is speculated that the cornea and lens of the patient did not develop in synchronization, resulting in a relatively wide corneoscleral transition zone with a normal size of the lens. This might be related to his genetic abnormality.

## 8. Conclusion

Herein, we report a challenging congenital cataract surgery in an adult case of colobomatous microphthalmia associated with intraorbital cyst. Phacoemulsification combined with implantation of an intraocular lens was performed in the right eye, without any intra- or postoperative complications. The patient was satisfied with the treatment and management. Clinically, patients with microphthalmia may experience a with other concurrent anomalies that can lead to more complicated conditions. Meticulous examination and evaluation and careful surgery can result in relatively good clinical outcomes.

## Data Availability

The data that support the findings of this study are available on request from the corresponding author. The data are not publicly available due to privacy or ethical restrictions.
