# Supplementary material for: Cataract Surgery in Congenital Colobomatous Microphthalmia Associated With Intraorbital Cyst in an Adult
Source: Case Rep Ophthalmol Med. 2025 Nov 10;2025:6625168. doi: 10.1155/crop/6625168 (PMC12623073; doi:10.1155/crop/6625168)
Supplement: Supporting Information — Additional supporting information can be found online in the Supporting Information section. The patient's family provided photographs of his life before and after surgery. [file 6625168.f1.zip › Supplementalfigure.docx]

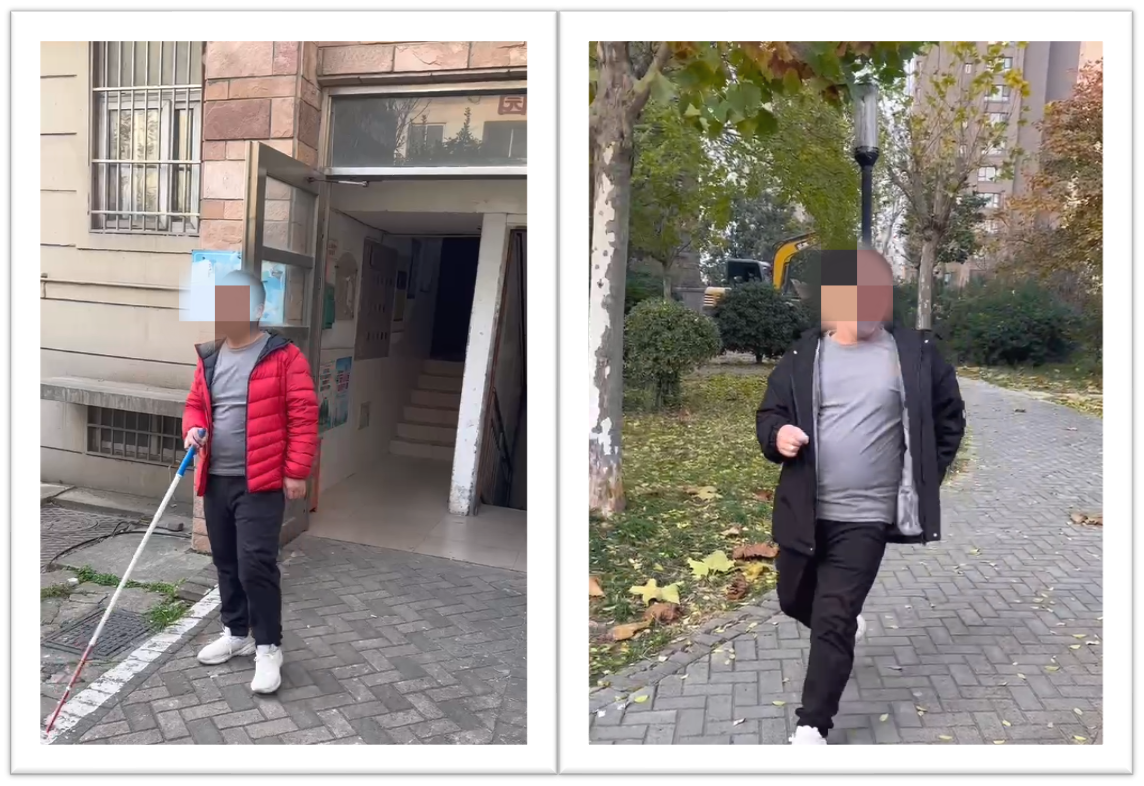


Supplemental figure 1. Patient-provided preoperative and postoperative photos during walking, demonstrating the marked improvement.


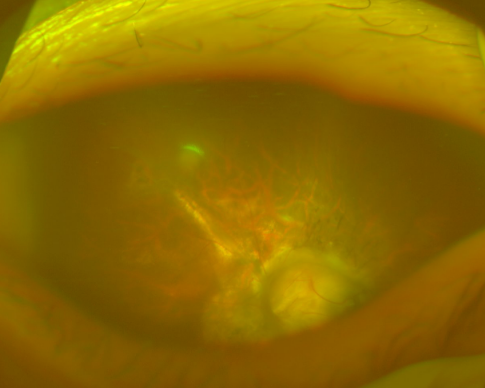


Supplemental figure 2. Ultra-widefield retinal imaging (Optos, UK) obtained at postoperative follow-up.
